# Exercise and Recovery Following Mild-to-Moderate Traumatic Brain Injury in the Community Setting

**DOI:** 10.7759/cureus.53459

**Published:** 2024-02-02

**Authors:** Edward J Weldon, Ryan W Nakamura, Tracy Van, Connor Goo, Anson Y Lee, Julia R Jahansooz, Enrique Carrazana, Kore K Liow

**Affiliations:** 1 Neurology, University of Hawaii John A. Burns School of Medicine, Honolulu, USA; 2 Neurology, Brain Research, Innovation & Translation Laboratory, Concussion and TBI Center, Concussion Research Lab, Hawaii Pacific Neuroscience, Honolulu, USA

**Keywords:** rehabilitation, recovery, exercise, concussion, traumatic brain injury

## Abstract

Introduction

The recommendations on return to exercise post-traumatic brain injury (TBI) remain debatable. As recent as 10 years ago, the conventional recovery modality for a mild TBI was to reduce neurostimulating activity and encourage rest until the symptoms subsided. However, emerging literature has challenged this notion, stating that returning to exercise early in the course of mild TBI recovery may be beneficial to the recovery timeline. This study surveys Hawaii’s diverse population to identify trends in exercise and recovery for TBI patients to shape recommendations on return to exercise.

Methods

A single-center retrospective chart review of the patients with mild-to-moderate TBI was selected from a patient database at an outpatient neurology clinic between January 2020 and January 2022. The variables collected include demographics, the etiology of injury, and symptoms at diagnosis. Self-generated phone surveys were completed to evaluate exercise patterns post-TBI.

Results

The patients who recovered within two years displayed similar exercise patterns to the patients who took more than two years to recover. Exercise frequency, intensity, and duration did not differ significantly (p=0.75, p=0.51, and p=0.80, respectively; n=100). Hiking and walking were more common in the long recovery (LR) group (p=0.02), likely reflecting advanced age compared to the short recovery (SR) group (50 versus 39 years, p<0.01). Additionally, no correlation exists between exercise intensity and worsening symptoms (p=0.920), suggesting that the patients exhibit exercise patterns suitable for sub-symptomatic recovery.

Conclusion

Return to exercise does not appear to be a predictor for mild-to-moderate TBI recovery. The patients appear to self-regulate an exercise regimen that will not exacerbate their symptoms or recovery time; thus, it may be suitable to recommend return to exercise as tolerated. These, and other findings in the literature, suggest that patients should be encouraged to return to exercise shortly after a mild TBI so long as the exercise does not exacerbate their symptoms.

## Introduction

Traumatic brain injury (TBI) is a significant cause of mortality and disability worldwide with an estimated 69 million people suffering from a TBI each year [1]. TBI is among the leading causes of disability particularly in those under 40 years old. Post-TBI conditions can significantly impact a person’s cognitive and locomotor functions, which negatively affects an individual’s autonomy [2]. There is an increasingly growing awareness surrounding the long-term cognitive and behavioral ramifications following a TBI, regardless of TBI severity. Although mild TBI has a relatively good prognosis within three months to one year, a repeated injury to the head during the recovery phase may exacerbate concussive symptoms and cause prolonged or even permanent cognitive, motor, or behavioral deficits [3]. Moderate-to-severe TBI can lead to incapacitating memory, executive function, mood, behavioral problems, and significant changes to cognition or behavior leading to heavy burdens on a patient’s social intelligence and family life. The multifactorial nature of TBI elicits a challenging treatment and management plan. The prognosis of a TBI is generally more favorable if the symptoms subside within the first six months after injury. The patients who regain full function following a TBI typically see symptoms subside within two years. However, if symptoms persist past this stage, the rate of improvement slows considerably. In some cases, the patients with persistent symptoms may see some extent of resolution five to 10 years later, but persistent TBI symptoms after two years pose a significant increase in complications, and the patient faces an uncertain prognosis [4]. Thus, it is of great interest to optimize the rate of recovery to achieve and preserve normal function following a TBI so that the patients can return to their normal daily functions.

The general recommendations for mild-to-moderate, uncomplicated post-TBI care involve medication management, increased rest, aversion to stimulating light or sound, psychotherapy, and routine follow-up [5]. However, post-TBI exercise recommendations remain a highly debated topic with long-standing literature and conventional approaches to recovery suggesting that return to contact sports and strenuous exercise should be avoided [6]. These studies assert that high-impact jarring exercises or intense aerobic exercises may exacerbate the patients’ post-TBI symptoms and delay recovery. On the other hand, emerging literature indicates that some level of mild-to-moderate exercise may actually enhance a patient’s recovery time. These new recommendations report that after a brief initial period of cognitive and physical rest (24-48 hours) following a TBI, the patients should be encouraged to gradually increase their activity [7-9]. Emerging data has revealed a significant shift in the approach of TBI management, arguing that sustained rest from stimulating cognitive and physical activity, the so-called “cocoon therapy,” does not benefit recovery [7,8]. The efficacy of timing, duration, and types of activity have not yet been clearly identified. Exercise has well-documented health benefits for all patients. By contributing to research that investigates the connection between TBI, exercise, and patient outcomes, we hope to improve TBI care and management to maximize the positive outcomes of TBI treatment.

The majority of this new research focuses on closely monitored, sub-symptom, aerobic treadmill exercise in a laboratory setting [8,10]. Although this recovery modality does have positive prognostic implications, its practicality is variable. Many of these studies that focus on a return to normal activity endpoint may be demographically condensed. These treadmill exercises may not be possible for patients who are elderly and morbidly obese, have concurrent lower body injuries, or lack the needed equipment. Furthermore, the recommendation of closely monitored, sub-symptom exercise requires demanding time and financial commitments from clinicians, patients, and family members, which may dissuade the patients from this recommendation. In this capacity, patients of the middle-to-lower class may not be able to access these new approaches to TBI care. This further illustrates the disparities between socioeconomic statuses and healthcare outcomes. Native Hawaiian and Pacific Islander (NHPI) populations are of particular interest because of their significant presence in Hawaii and the observation that they have been historically linked to disproportionate healthcare disparities [11]. Because each TBI has a unique etiology, pathophysiology, and numerous confounding variables, such as the patient’s current health, a generalized recommendation for efficacious recovery is not yet well-defined [12]. Therefore, this research aims to study self-directed exercise patterns and modalities following mild-to-moderate TBI in Hawaii’s demographically diverse community setting to shape recommendations on return to exercise, identify suitable exercises, and characterize potential barriers to recovery.

This article was previously presented as a meeting abstract at the 2023 American Academy of Neurology (AAN) Annual Meeting on April 25, 2023.

## Materials and methods

**Figure 1:**
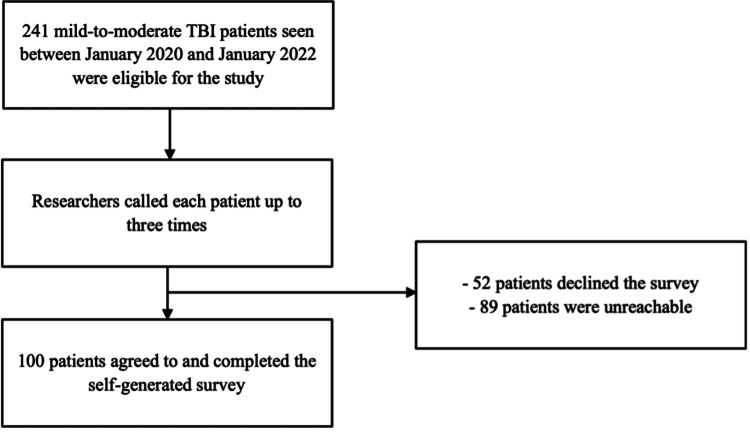
PRISMA Flowchart of the Inclusion Criteria PRISMA, Preferred Reporting Items for Systematic Reviews and Meta-Analyses; TBI, traumatic brain injury

A single-centered, retrospective chart review was conducted to identify the patients diagnosed with TBI in Hawaii between January 2020 and January 2022. The patients were initially screened for a history of TBI based on their International Classification of Diseases, Tenth Revision (ICD-10) code for concussion with or without the loss of consciousness (S06.0X, with codes containing various fifth, sixth, and seventh digits) for the two-year period by a team of board-certified neurologists. To select a cohort of patients with mild-to-moderate TBIs, electronic medical records of patients were accessed, as well as those found to have abnormal structural imaging, loss of consciousness for >24 hours, severe alteration of consciousness or mental state for >24 hours, post-traumatic amnesia greater than seven days, or Glasgow Coma Scale (GCS) of less than 9, and characteristics of severe TBIs, and the patients who had ongoing symptoms for less than two years were excluded from the analysis [12]. Following the exclusion criteria, 241 patients were eligible for the study and phone surveys. A self-generated phone survey was then administered to eligible patients to evaluate symptom duration, recovery methods, employment, perceived barriers to recovery, exercise patterns post-TBI, and perceptions of exercise and recovery. Of the 241 eligible patients, 100 patients agreed to complete the self-generated phone survey (Figure 1).

Upon the randomization of phone call assignments, the researchers called the patients up to a maximum of three times before excluding the patient from the study. Fifty-two patients declined the survey due to a variety of reasons, and 89 were unreachable. A total of 100 patients agreed to and completed the questionnaire; thus, these completed questionnaire responses were included for statistical analysis. Survey contents included questions regarding the patient’s perception of recovery status, current symptomatology, employment status prior to TBI, current employment status, changes to employment status, recovery strategies, perceived barriers to recovery (i.e., work, and finances), and education level. Additional questions focused on the patient’s exercise regimen. The questions included the patient’s frequency of exercise, modality of exercise, and intensity of exercise before and after the TBI and whether the post-TBI exercise improved, worsened, or did not change any TBI symptoms.

Full electronic medical records were then reviewed following survey completion. The variables collected from the chart review include demographics, etiologies, and symptoms at diagnosis. Based on reported recovery time and medical records, the patients were divided into two groups for analysis based on outcomes [2]: the short recovery (SR) group who fully recovered within two years of TBI and the long recovery (LR) group who did not recover fully within two years. Following data collection through phone surveys and retrospective chart review, data analysis was performed using RStudio (Posit PBC, Boston, MA).

Categorical variables were analyzed using Pearson’s chi-squared test and Fisher’s exact test. Continuous variables were analyzed using the Wilcoxon rank sum test.

## Results

A description of the study sample is presented in Table 1. The LR group, listed as “two or more years,” was significantly older than the SR group at the time of diagnosis (50 versus 39 years old, p=0.003). Additionally, there were significant differences in employment status between the two groups (p=0.027), with fewer LR patients employed compared to SR (30.2% versus 51.4%), more LR patients unemployed (34.9% versus 18.9%), and more LR patients retired (28.6% versus 16.2%). Apart from that factor, sex, race, BMI, insurance type, marital status, education level, a history of depression, a history of TBI, smoking status, drinking status, and cannabis use status did not differ among the two groups.

**Table 1:** Patient Demographics ^1^Mean (SD); n (%) ^2^Wilcoxon rank sum test; Pearson’s chi-squared test; Fisher’s exact test NHPI, Native Hawaiian and Pacific Islander; TBI, traumatic brain injury

Variables	Less than two years (N=37)^1^	Two or more years (N=63)^1^	Overall (N=100)^1^	p-value^2^
Age	43 (18.5)	55 (14.6)	51 (17.1)	<0.001
Sex				0.2
Male	19 (51.4%)	24 (38.1%)	43 (43.0%)	
Female	18 (48.6%)	39 (61.9%)	57 (57.0%)	
Race				0.26
White	16 (43.2%)	28 (44.4%)	44 (44.0%)	
Asian	12 (32.4%)	11 (17.5%)	23 (23.0%)	
NHPI	7 (18.9%)	15 (23.8%)	22 (22.0%)	
Other race	2 (5.4%)	9 (14.3%)	11 (11.0%)	
BMI	28 (8.3)	28 (7.6)	28 (7.8)	0.68
Insurance type				0.37
Medicare	4 (10.8%)	13 (20.6%)	17 (17.0%)	
Medicaid	12 (32.4%)	20 (31.7%)	32 (32.0%)	
Private	20 (54.1%)	25 (39.7%)	45 (45.0%)	
Military	1 (2.7%)	5 (7.9%)	6 (6.0%)	
Marital status				0.54
Unmarried	20 (54.1%)	38 (60.3%)	58 (58.0%)	
Married	17 (45.9%)	25 (39.7%)	42 (42.0%)	
Education level				>0.99
Did not complete high school	1 (2.7%)	3 (4.8%)	4 (4.0%)	
High school diploma	15 (40.5%)	25 (39.7%)	40 (40.0%)	
College degree	16 (43.2%)	26 (41.3%)	42 (42.0%)	
Graduate degree	5 (13.5%)	9 (14.3%)	14 (14.0%)	
Current employment status				0.027
Employed	19 (51.4%)	19 (30.2%)	38 (38.0%)	
Unemployed	7 (18.9%)	22 (34.9%)	29 (29.0%)	
Retired	6 (16.2%)	18 (28.6%)	24 (24.0%)	
Medical leave	1 (2.7%)	1 (1.6%)	2 (2.0%)	
Student	4 (10.8%)	1 (1.6%)	5 (5.0%)	
Disabled	0 (0.0%)	2 (3.2%)	2 (2.0%)	
History of prior TBI	11 (29.7%)	16 (25.4%)	27 (27.0%)	0.64
Age at diagnosis of TBI	39 (20.0)	50 (15.5)	46 (18.0)	0.003
Diagnosis of depression at presentation of TBI	10 (27.0%)	16 (25.4%)	26 (26.0%)	0.86
Diagnosis of depression at most recent clinic visit	11 (29.7%)	10 (15.9%)	21 (21.0%)	0.1
Smoker at most recent clinic visit	8 (21.6%)	7 (11.1%)	15 (15.0%)	0.16
Drinker at most recent clinic visit	10 (27.0%)	17 (27.0%)	27 (27.0%)	>0.99
Cannabis user at most recent clinic visit	5 (13.5%)	4 (6.3%)	9 (9.0%)	0.28

TBI etiologies and symptoms at diagnosis are presented in Table 2. “Fall” was the most common etiology of TBI among both patient groups at 37%, followed by “motor vehicle accident” (MVA) at 30%. When comparing the SR and LR groups, there were no significant differences found in the cause of TBI (p=0.93). Additionally, there were no significant differences in symptom presentation between the two groups including migraines or headaches (p=0.87); dizziness, lightheadedness, nausea, or vomiting (p=0.70); balance issues (p=0.56); memory changes (p=0.68); amnesia (p=0.91); psychiatric symptoms at diagnosis (p=0.57); the loss of consciousness (p=0.94); or hospitalization (p=0.67).

**Table 2:** Summary of the TBI Symptoms of the Study Population ^1^Mean (SD); n (%) ^2^Wilcoxon rank sum test; Pearson’s chi-squared test TBI, traumatic brain injury; MVA, motor vehicle accident

Variables	Less than two years (N=37)^1^	Two or more years (N=63)^1^	Overall (N=100)^1^	p-value^2^
Cause of TBI				0.93
Fall	13 (35.1%)	24 (38.1%)	37 (37.0%)	
MVA	10 (27.0%)	20 (31.7%)	30 (30.0%)	
Assault	2 (5.4%)	4 (6.3%)	6 (6.0%)	
Sports	4 (10.8%)	5 (7.9%)	9 (9.0%)	
Others	8 (21.6%)	10 (15.9%)	18 (18.0%)	
Presence of migraines or headaches	28 (75.7%)	46 (74.2%)	74 (74.0%)	0.87
Dizziness, lightheadedness, nausea, or vomiting	15 (40.5%)	28 (44.4%)	43 (43.0%)	0.7
Balance issues	12 (32.4%)	17 (27.0%)	29 (29.0%)	0.56
Change in memory	12 (32.4%)	23 (36.5%)	35 (35.0%)	0.68
Psychiatric symptoms at diagnosis	7 (18.9%)	15 (23.8%)	22 (22.0%)	0.57
Loss of consciousness	22 (59.5%)	37 (58.7%)	59 (59.0%)	0.94
Hospitalized	16 (43.2%)	30 (47.6%)	46 (46.0%)	0.67
Amnestic to the event	5 (13.5%)	9 (14.3%)	14 (14.0%)	0.91

A summary of patient recovery regarding therapies utilized and exercise characteristics comparing the SR and LR groups is found in Table 3. Despite employment change discrepancies between the SR and LR groups, there was no significant difference in weeks taken off from work (4±8.5 and 4±7.3, p=0.13). SR patients were significantly more likely to utilize increased rest for recovery (54.1% versus 25.4%, p<0.01). Apart from increased rest, there were no significant differences in therapies utilized, including traditional medicine, alternative medicine, light aversion, decreased screen time, psychotherapy, or rehabilitation/physical therapy. Regarding exercise modalities utilized during TBI recovery, the LR group was significantly more likely to utilize walking (81.8% versus 58.8%, p=0.02). Otherwise, there were no significant differences in exercise modality utilization including resistance training, running, swimming, biking, basketball, martial arts, and bodyweight fitness classes.

**Table 3:** Descriptive Summary of the Patients’ TBI Recovery by Symptom Duration ^1^Mean (SD); n (%) ^2^Wilcoxon rank sum test; Pearson’s chi-squared test; Fisher’s exact test OTC, over-the-counter; Rx, prescription; TBI, traumatic brain injury

Variables	Less than two years (N=37)^1^	Two or more years (N=63)^1^	Overall (N=100)^1^	p-value^2^
Time off from work (in weeks)	4 (8.5)	4 (7.3)	4 (7.8)	0.13
Treatment				
Traditional medicine (OTC, Rx)	6 (16.2%)	20 (31.7%)	26 (26.0%)	0.087
Alternative medicine	4 (10.8%)	10 (15.9%)	14 (14.0%)	0.48
Increased rest	20 (54.1%)	16 (25.4%)	36 (36.0%)	0.004
Light aversion	7 (18.9%)	5 (7.9%)	12 (12.0%)	0.12
Decreased screen time	6 (16.2%)	6 (9.5%)	12 (12.0%)	0.35
Psychotherapy	4 (10.8%)	10 (15.9%)	14 (14.0%)	0.48
Rehabilitation or physical therapy	10 (27.0%)	27 (42.9%)	37 (37.0%)	0.11
Others	10 (27.0%)	13 (20.6%)	23 (23.0%)	0.46
Exercise				
Resistance training	11 (32.4%)	12 (21.8%)	23 (23.0%)	0.27
Running	9 (26.5%)	11 (20.0%)	20 (20.0%)	0.48
Swimming/paddling	9 (26.5%)	12 (21.8%)	21 (21.0%)	0.62
Biking	6 (17.6%)	7 (12.7%)	13 (13.0%)	0.55
Basketball	1 (2.9%)	1 (1.8%)	2 (2.0%)	>0.99
Surfing	0 (0.0%)	3 (5.5%)	3 (3.0%)	0.28
Martial arts	2 (5.9%)	3 (5.5%)	5 (5.0%)	>0.99
Bodyweight fitness class	10 (29.4%)	10 (18.2%)	20 (20.0%)	0.22
Hiking/walking	20 (58.8%)	45 (81.8%)	65 (65.0%)	0.018
Others	9 (26.5%)	6 (10.9%)	15 (15.0%)	0.057
Exercise intensity				0.51
Mild	17 (50.0%)	33 (60.0%)	50 (50.0%)	
Moderate	16 (47.1%)	19 (34.5%)	35 (35.0%)	
Intense	1 (2.9%)	3 (5.5%)	4 (4.0%)	
Days of exercise per week (mean)	4 (1.4)	4 (1.7)	4 (1.6)	0.75
Average workout length				0.8
0-<60 minutes	17 (50.0%)	28 (50.9%)	45 (45.0%)	
60-<90 minutes	11 (32.4%)	20 (36.4%)	31 (31.0%)	
90+ minutes	6 (17.6%)	7 (12.7%)	13 (13.0%)	
Improved symptom change with exercise	22 (64.7%)	33 (60.0%)	55 (550%)	0.66
Worsened symptom change with exercise	10 (29.4%)	16 (29.1%)	26 (26.0%)	0.97
No symptom change with exercise	10 (29.4%)	14 (25.5%)	24 (24.0%)	0.68
Perceived barriers to recovery				
Stress	12 (32.4%)	19 (30.2%)	21 (21.0%)	0.81
Work or school	11 (29.7%)	14 (22.2%)	25 (25.0%)	0.4
Finances	10 (27.0%)	23 (36.5%)	33 (33.0%)	0.33
Lack of support	6 (16.2%)	10 (15.9%)	16 (16.0%)	0.96
Physical barriers	4 (10.8%)	10 (15.9%)	14 (14.0%)	0.48
Family or children	4 (10.8%)	4 (6.3%)	8 (8.0%)	0.46

Regarding exercise patterns, there were no significant differences in exercise frequency, exercise intensity, and workout duration (p=0.75, p=0.51, and p=0.80). Furthermore, no significant differences were found between the duration of TBI symptoms and the patient’s symptom change with exercise. Of patients in the SR and LR groups that worked out, 29.4% and 29.1% reported worsened symptoms with exercise, and the rest either reported no change or improved symptom profile (p=0.48). Table 3 also includes data on perceived barriers to recovery among the SR and LR groups. Finances were the most cited barrier to recovery in the LR group at 36.5%, and stress was the most cited barrier in the SR group at 32.4%. Nevertheless, perceived barriers to recovery were not significant predictors for long versus short recovery.

Finally, when stratifying symptom change during exercise based on exercise intensity shown in Table 4, exercise intensity was not a significant predictor of whether a patient would experience worsening symptoms during exercise. Additionally, most patients across the study expressed satisfaction with their decision to exercise following TBI (90.9%), and nearly all patients said that they would recommend exercise to others recovering from a TBI (98.9%).

**Table 4:** Symptom Change by the Patients’ Exercise Intensity ^1^n (%) ^2^Fisher’s exact test

Variables	Mild (N=50)^1^	Moderate (N=35)^1^	Intense (N=4)^1^	p-value^2^
Improved symptom change with exercise	28 (56.0%)	24 (68.6%)	3 (75.0%)	0.48
Worsened symptom change with exercise	14 (28.0%)	11 (31.4%)	1 (25.0%)	0.92
No symptom change with exercise	15 (30.0%)	8 (22.9%)	1 (25.0%)	0.84

## Discussion

Emerging research focusing on recovery recommendations for TBI continues to shed new light on conventional literature and approaches. We believe that the data presented in this study may contribute to a better understanding of patient exercise characteristics following TBI and will help to inform physicians of the risks and benefits of allowing patients to exercise on their own.

For the most part, demographics were quite similar between the SR and LR groups (Table 1). The first demographic that was found to be different between the two groups was age, as the LR group was significantly older than the SR group. Previous studies have definitively shown that advanced age is associated with both prolonged and potentially incomplete recovery, so the finding that the LR group is significantly older is to be expected [13]. The patients who took two or more years to recover were significantly less likely to be employed and more likely to be unemployed. Advanced age in the LR group likely contributed to a higher percentage of retired patients but was unlikely to fully explain the changes in employment status. Regardless of age, TBI has been found to have significant detrimental effects on the ability to work and, as a result, employment status. Thus, our findings were consistent with existing literature [14]. We believe that these findings demonstrate that the SR and LR groups were demographically comparable. The further analysis of clinical presentation among these two groups (Table 2) showed no differences in etiology and clinical presentation at the time of diagnosis. The strong similarities between etiologies and symptoms at diagnosis suggested that the TBIs sustained by the patients in both groups were similar, so comparing recovery outcomes between the SR and LR groups is appropriate.

This study found no significant correlation between time-to-exercise, exercise modalities, frequencies, durations, and intensities with respect to recovery time, suggesting that exercise following TBI was not a significant predictor of recovery time.

Previous studies that examined exercise post-TBI focused on treadmill training, which, while convenient for laboratory observation, may not be preferable for many [8]. This study aimed to identify potential alternative exercises that could be completed in a community setting that would garner the benefits of exercise without the potential negatives of symptom exacerbation and prolonged recovery. When examining resistance training, running, swimming, biking, basketball, martial arts, and bodyweight fitness classes, there was no correlation between exercise modality and recovery time, suggesting that a wide variety of exercises may be suitable for recovering patients. Interestingly, the only significant correlation between exercise type and recovery time was walking, which was disproportionately represented in the LR group. Walking is generally considered to be among the least physically demanding exercises available, so at first appearance, it may seem odd to be commonly performed with LR patients. However, this finding is likely attributable to the advanced age of the LR group. Walking is often the preferred exercise of older individuals over more orthopedic-demanding sports and may be the most strenuous exercise appropriate for their level of fitness or physical ability. An alternative explanation is that due to TBI severity, exercise intensity capacity was limited resulting in higher rates of walking. However, this explanation is unlikely given the similar symptom profile between the LR and SR groups and the lack of correlation between recovery time and exercise intensity described in Table 2.

A common misconception for returning a patient to exercise following a TBI is that highly strenuous activities may increase the risk of exacerbating headaches and other symptoms leading to prolonged recovery time. However, our study showed no correlation between exercise intensity and recovery time, exercise intensity and resulting symptom change, or symptom change with exercise and recovery time. As a whole, the majority of patients were able to tolerate some level of exercise without worsening any symptoms, though exercise intensity did vary from person to person. We believe that the patients were self-regulating exercise regimens to generally remain sub-symptomatic and, as a result, not prolong recovery. Previous literature has shown that sub-symptomatic exercise in a laboratory setting following a TBI is potentially beneficial, and our findings suggested that the same may be true for exercise in an uncontrolled community setting [8]. These findings are further strengthened by the fact that the vast majority of patients expressed satisfaction with their decision to exercise post-TBI and recommended that other recovering individuals exercise.

Finally, the inclusion of NHPI patients was useful in discovering potential disparities in recovery outcomes and resources for this racial group. This study incidentally discovered worrying disparities that warrant future research into recognizing these trends and identifying potential solutions. A variety of resources are necessary to consider when recovering from a TBI. It is generally understood that limiting neurocognitive stimuli during the recovery phase is important for safe and efficacious rehabilitation [15]. Alternative medicine, psychotherapy, physical rehabilitation, and pharmaceutical intervention may also be applicable to aid in recovery. Racial minorities in Hawaii, including NHPI, subjectively reported accessing these resources at substantially lower rates than White patients in our study cohort. The speculative mechanism behind this disparity may reside in the widened socioeconomic status gap between these two racial groups. NHPI individuals have long been identified as a racial group that has disproportionate adverse health outcomes and limited access to health resources in Hawaii [11]. These disparities were similarly reflected in our study. Although further studies with a larger sample size would be beneficial to significantly verify such trends, the current results were consistent with existing literature that explores racial and ethnic disparities across the United States, particularly in Asian and Hispanic groups [16]. Perhaps the most meaningful component of this lack of resource access was the inability to accommodate an appropriate treatment plan when factoring in social and economic considerations. Lower-to-middle-class individuals may not be able to afford the necessary time off from work or other duties to properly recover from their TBIs. They would be less inclined to pay for and dedicate time to pursuing additional therapeutic interventions that favor faster and better recoveries. In an increasingly diverse US population, return to exercise recommendations should also consider a patient’s racial, cultural, and psychosocial background, as well as their medical literacy and ability to access the most suitable recovery regimen. With a newfound appreciation for modern approaches to mild-to-moderate TBI recovery, there is considerable work to be done to address the psychosocial aspects of TBI care that are likely to manifest. Further investigation should evaluate perceived physical, social, and financial barriers to TBI care to better understand how to approach these disparities from a policy change level.

This study had several limitations. Our research only included the patients with TBIs seen at an outpatient neuroscience clinic, meaning that they possibly suffered TBIs of greater than average severity, limiting the generalizability of the study. The patients who suffered mild TBI with short recovery times or due to seemingly trivial head injuries are more likely to remain solely under the care of their primary care physicians; thus, these patients may not have been captured in our data set. The study is also limited by its sample size. There are several, plausible features of the LR group that may have contributed to a longer recovery time. However, this was not captured in our statistical analysis, which may reflect a possible type 1 error. This may warrant further investigation with a larger sample size to increase the power of the study. Additionally, retrospectively surveying patients may induce recall bias. However, this risk was minimized by only collecting data on TBIs occurring since 2020.

## Conclusions

Return to exercise following TBI in the general population does not appear to be a predictor for recovery time. Though the potential danger of symptom exacerbation with exercise remains a valid concern, the lack of correlation between exercise patterns and recovery time and the lack of correlation between exercise patterns and corresponding symptom changes suggest that patients who can safely self-regulate their exercise to prevent symptom exacerbation will not prolong their recovery time. As a result, we believe that encouraging the patients to return to exercise as tolerated following a TBI will not exacerbate the duration or symptoms of TBI recovery. Nevertheless, the patients should still be cautioned about the dangers of symptom exacerbation and prolonged recovery and encouraged to avoid high-impact activities. Additionally, more attention should be paid to addressing barriers to access to TBI recovery resources, especially for underserved racial groups.
